# Improving clinical diagnosis of early-stage cutaneous melanoma based on Raman spectroscopy

**DOI:** 10.1038/s41416-018-0257-9

**Published:** 2018-11-09

**Authors:** Inês P. Santos, Remco van Doorn, Peter J. Caspers, Tom C. Bakker Schut, Elisa M. Barroso, Tamar E. C. Nijsten, Vincent Noordhoek Hegt, Senada Koljenović, Gerwin J. Puppels

**Affiliations:** 1 Department of Dermatology, Erasmus MC, Erasmus University Medical Center Rotterdam, Rotterdam, Netherlands; 2 Department of Dermatology, Leiden University Medical Center, Leiden, Netherlands; 3 Department of Oral & Maxillofacial Surgery, Special Dental Care, and Orthodontics, Erasmus MC, Erasmus University Medical Center Rotterdam, Rotterdam, Netherlands; 4 Department of Pathology, Erasmus MC, Erasmus University Medical Center Rotterdam, Rotterdam, Netherlands

## Abstract

**Background:**

Clinical diagnosis of early melanoma (Breslow thickness less than 0.8 mm) is crucial to disease-free survival. However, it is subjective and can be exceedingly difficult, leading to missed melanomas, or unnecessary excision of benign pigmented skin lesions. An objective technique is needed to improve the diagnosis of early melanoma.

**Methods:**

We have developed a method to improve diagnosis of (thin) melanoma, based on Raman spectroscopy. In an ex vivo study in a tertiary referral (pigmented lesions) centre, high-wavenumber Raman spectra were collected from 174 freshly excised melanocytic lesions suspicious for melanoma. Measurements were performed on multiple locations within the lesions. A diagnostic model was developed and validated on an independent data set of 96 lesions.

**Results:**

Approximately 60% of the melanomas included in this study were melanomas in situ. The invasive melanomas had an average Breslow thickness of 0.89 mm. The diagnostic model correctly classified all melanomas (including in situ) with a specificity of 43.8%, and showed a potential improvement of the number needed to treat from 6.0 to 2.7, at a sensitivity of 100%.

**Conclusion:**

This work signifies an important step towards accurate and objective clinical diagnosis of melanoma and in particular melanoma with Breslow thickness <0.8 mm.

## Introduction

Cutaneous melanoma is a malignant tumour arising from melanocytes, the pigment-producing cells. It is one of the most aggressive and fatal forms of skin malignancy. Its incidence has been steadily increasing in the last decades, with 351,880 new cases in 2015.^1^

Diagnosis of melanoma at an early stage is desirable. One of the most important prognostic factors of melanoma is the vertical depth of growth (Breslow thickness). The Breslow thickness is significantly correlated with metastatic propensity. Melanoma in situ has no associated direct mortality but carries the risk of progressing to an invasive stage.^2^ Melanoma with a Breslow thickness less than 0.8 mm can be treated surgically with a high cure rate (5-year survival rate of >97%).^2^ In advanced stages, the 5-year survival rate is reported to be approximately 30%.^3^

The clinical diagnosis of melanoma is based on analysis of morphological criteria and is therefore, subjective and can be difficult for general practitioner as well as dermatologist. It is performed by visual inspection of the lesion, aided by dermoscopy. When a lesion is clinically suspected of melanoma, a diagnostic excision is indicated.

It has been reported that among general practitioners, the sensitivity of diagnosing melanoma varies between 70 and 88% and among dermatologists between 82 and 100%.^4^ However, the number needed to treat (NNT, the number of benign pigmented lesions excised to detect one melanoma), varies between 6.3 and 8.7 by dermatologists,^5,6^ and between 20 and 30 for general practitioners.^5–13^ The NNT can be even higher in a population of patients <30 years (NNT = 75)^7–9^ or in high-risk populations (NNT = 34, e.g. multiple dysplastic nevi or familial melanoma).^10^

These numbers imply that melanomas can be clinically missed, with the risk of missing the opportunity to cure the patient, while many unnecessary excisions of benign lesions take place. An objective and easy-to-use technique that will support and improve the clinical diagnosis of thin melanoma is needed to complement the still limited diagnostic toolbox in current dermatological practice.

The reported efforts to develop techniques to improve the clinical diagnosis of melanoma are promising. Nevertheless, detecting early-stage melanomas is challenging. Most methods rely on the detection of morphological differences between benign and malignant pigmented skin lesions.^11–21^ Several studies show improvement in diagnostic accuracy of morphology-based methods combined with dermoscopy. Yet most of these techniques are operator dependent and subject to interpretation.^12,13^ Using confocal microscopy in an in vivo setting, Monheit et al. reported a sensitivity of 98.4% and a specificity of 9.9% with an independent validation set.^22^ A recent pilot study conducted by Delpueyo et al. on multispectral imaging showed a sensitivity of 87.2% and specificity of 54.5% with an independent validation set.^17^ Using in vivo reflectance confocal microscopy in clinically suspicious lesions, Alarcon et al. reported a sensitivity of 97.8% and a specificity of 92.4%, with six melanoma in situ missed.^12^ The presented results are promising; however, reflectance confocal microscopy is dependent on the experience of the operator.^23^ In a multicentre study, 1300 lesions were analyzed by electrical impedance spectroscopy to discriminate melanoma from benign lesions: the observed sensitivity was 99.4%, and the specificity was 35.5% when dysplastic nevi were excluded, or 23.9% when dysplastic nevi were included.^20^ In another multicentre study, 2416 lesions were analyzed by electrical impedance spectroscopy for melanoma detection. Sensitivity of 96.6% and a specificity of 34.4% were reported.^24^

Compared to the morphology, biochemical tissue characteristics are more specific.^25–27^ Raman spectroscopy is an optical nondestructive technique that goes beyond morphology analysis and characterises the tissue at a molecular level. It has been amply demonstrated that Raman spectra can be used to distinguish cancer from healthy tissue, including pigmented skin lesions.^28–33^

Lui et al. developed a classification model to distinguish benign pigmented lesions from melanoma, using a large Raman measurement volume.^32^ Only 28% of the clinically benign lesions were histopathologically confirmed.^32^ The authors included in vivo Raman measurements acquired from 44 melanomas and 286 clinically benign pigmented skin lesions. The authors reported sensitivity of 99% and specificity of 15%. This system, Aura®-system (Verisante, Canada), for skin cancer detection was commercialised. In a more recent study, the same authors increased the number of lesions (only nine melanomas were added) in the same clinical setting and performed an independent validation.^33^ In this study, the sensitivity was 99% and the specificity was 24%.

Our group has previously demonstrated the feasibility to acquire high-quality Raman spectra of pigmented tissue samples in the short-wave infrared (SWIR) region.^34^ The study was performed in a tertiary referral centre for high-risk patients (familial melanoma, previous melanoma). All lesions suspicious for melanoma based on the evaluation by specialised dermatologists aided by dermoscopy were excised for histopathological diagnosis. In that study, we measured 124 freshly excised melanocytic lesions. In some cases (*n* = 42) there was an uneven distribution of histological components throughout the lesion, leading to the possible sampling of nonmelanocytic tissue (e.g. dermal collagen or skin appendages); these were referred to as histopathologically heterogeneous lesions. Because for heterogeneous lesions no accurate point-to-point correlation between the locations of the individual Raman measurements and individual histological components could be made, these were not used for the development of a classification model. Therefore, the model was limited to histopathologically homogeneous lesions, which resulted in a specificity of 45% and a sensitivity of 100%.^31^ This confirms that there is spectroscopic information in the 2820−3040 cm^−1^ region (assigned to CH_2_−CH_3_ stretching vibrations), which can be used to discriminate melanomas from benign melanocytic lesions. The results showed that the most distinctive spectral feature can be attributed to a higher lipid−protein ratio in melanomas.^31^

In this paper, we have addressed the challenge of representative Raman sampling of melanocytic lesions. We have developed a Raman spectroscopy method to distinguish between melanoma and not-melanoma irrespective of the histopathological heterogeneity of the lesions. The fundamental requirement of the diagnostic model was to not miss any melanoma (100% sensitivity). The diagnostic model was validated on an independent data set.

## Materials and methods

### Specimen handling

**Fig. 1:**
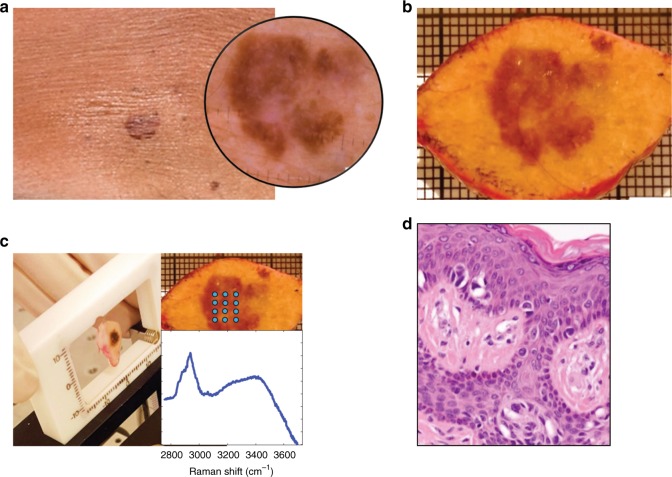
Schematic representation of specimen handling. **a** Clinical diagnosis was aided by dermoscopy (inset). **b** Lesions suspicious for melanoma were excised. **c** Specimen is inserted in the cartridge and multiple points were measured within the lesion (inset). **d** Routine histopathological evaluation (image from H&E slide)

This study was approved by the Medical Ethics Committee of the Leiden University Medical Center (LUMC) (C13.06). After clinical assessment performed by a dermatologist, pigmented skin lesions clinically suspicious for melanoma were excised according to the national melanoma guideline and standard protocol of the LUMC Department of Dermatology. Immediately after surgery, the specimens were prepared for Raman spectroscopy measurements. They were rinsed with NaCl solution (0.9%), wiped with a gauze soaked in ethanol (70%, to remove residual ink from pen marker), gently flattened between two fused silica windows and inserted into a custom-made specimen cartridge for Raman measurements, as illustrated in Fig. 1. For detailed description, see our previous study.^31^ After the Raman spectroscopy measurements, the skin specimens were emerged in a 4% formaldehyde solution and sent to pathology department for the routine diagnostic procedure.

### Raman spectroscopy measurements

Raman measurements were performed using an SWIR multichannel Raman instrument, which records in the spectral range 2780−3750 cm^−1^. This instrument was constructed in-house and has been described previously.^34^ The light source was a diode laser with a wavelength of 976 nm (IPS, Monmouth Junction, NJ, USA). The light was focused on the skin lesion to a spot with a diameter of ~6 μm. Per lesion, an average of 14 (range 9–19) point measurements were performed in the pigmented region of the excised lesion. Each point measurement had an integration time of 30 s.

#### Calibration

All Raman spectra were corrected for the wavelength-dependent detection efficiency of the instrument, using an SRM2246 intensity standard (National Institute of Standards and Technology, Gaithersburg, MD), as explained elsewhere.^34^ The absolute wavenumber axis was calibrated using the spectral lines of a neon−argon lamp and the Raman spectrum of cyclohexane. The Raman background that originates from the optics was subtracted from all spectra. Data were filtered by fifth-order spline filter to remove fixed pattern noise.^31^

#### Reference spectra

As reference, Raman spectra were measured from collagen type I (Sigma-Aldrich, C7774—CAS Number 9007-34-5). For the keratin, Raman spectra were measured in vivo from the thick stratum corneum on the sole of the foot of a healthy volunteer.

Moreover, a set of reference spectra from tissue with low fluorescence background and high variance of Raman signal was created from the spectra of skin lesions used in this study, as follows. For each spectrum, the ratio between peak content and background content was calculated using the spectral region: 2830−3020 cm^−1^ (CH_3_ stretching vibrations, which are abundantly present in all biological tissues). A first-order polynomial baseline was fitted through the spectral points at 2830 and 3020 cm^−1^. Peak content was calculated as the integrated area above this baseline. Background was calculated as the integrated area below the baseline. The spectra with the highest 40% peak to background ratio values were selected and divided into 20 clusters using hierarchical cluster analysis. Only clusters that consisted of more than five spectra were selected, and the spectral average per cluster was calculated. The resulting 17 cluster average spectra were considered as an HWVN tissue reference spectral library.

### Preprocessing of Raman spectra

All Raman spectra were pre-processed in the way described below. The software used for all computations in this study was Matlab R2015b (Mathworks Inc., Natick, MA).

#### Tissue background subtraction

A method described by Barroso et al. based on multiple regression fitting (MRF), was used for background correction.^35^ MRF is an unsupervised method that corrects fluorescence spectra independently of the shape and intensity of the Raman signal. Briefly, a set of background-free library spectra and a second-order polynomial were fitted to the data using a non-negative least squares method. As the library spectra describe all Raman variance present in the data with minimal background signal, the fitted polynomial is a good approximation of the fluorescence background present in the data. The approximated backgrounds were subtracted from the respective spectra.

#### Correction for variations in the water signal

A similar procedure was performed to remove the influence of water signal in the CH band. A reference spectrum of water was fitted to the data. The coefficient that better approximated the water reference spectrum for each one of the spectra measured was obtained. The water signal multiplied by the corresponding coefficient was subtracted from the correspondent data spectra.

#### Scaling

All the spectra were scaled to the average of all individual spectra using an extended multiplicative signal correction with a zero-order polynomial background^36^ and cropped to the spectral range (2800−3050 cm^−1^), which corresponds to the CH-stretching band region.

#### Detection of spectrally heterogeneous lesions

The spectral variance within each lesion was calculated (in the complete range 2800−3050 cm^−1^). The spectral variance per lesion was then added in the spectral direction, to have a total variance. The variance of each lesion was normalised with respect to the maximum variance. The lesions with a high variance (in the top 10% of the ranked values) were considered spectrally heterogeneous and were added to the heterogeneous lesions group.

#### Signal orthogonalization for keratin and collagen

A Raman spectrum obtained from a melanocytic lesion can include contributions from several nonmelanocytic tissue components as mentioned above (e.g. collagen from dermis and keratin from stratum corneum). These contributions are not informative for the discrimination of melanocytic lesions. A method described by Maquelin et al.^37^ was used to estimate the Raman signal variance in the spectra from keratin or from collagen. The method is based on a mathematical projection of Raman lesion spectrum on reference spectra of keratin or collagen. The results yield the Raman signal that cannot be distinguished from the signal of keratin or collagen. Subsequent subtraction of this projection from the spectrum results in the desired nonkeratin or noncollagen-related Raman lesion spectra (i.e. the vector component of the spectrum that is orthogonal to keratin or collagen).

In a first step, all Raman spectra obtained from lesions were orthogonalised with the reference spectrum of keratin. After projection and subsequent subtraction, the integrated absolute intensity of the orthogonalised spectrum was calculated. These intensities were normalised to the mean value. Given the fact that the spectra that have a low integrated absolute intensity cannot be distinguished from the spectra of keratin, it was assumed that the measurement was performed in a region with a thick overlying stratum corneum. Spectra of which the integrated intensity was below the threshold defined at 0.68 (i.e. high presence of keratin) were discarded. These Raman spectra were dominated by keratin and considered not suitable for classification. Orthogonalised spectra on keratin that presented an integrated area lower than 0.68 (i.e. high presence of keratin) were labeled as “Not predicted”.

In a second step, the Raman spectra were orthogonalised with the reference spectrum of collagen and integrated. The integrated absolute intensities were normalised to the mean value. Lesion spectra with an integrated intensity lower than 0.56, which means that the original spectra had high contribution of collagen, were considered to be originated from nonmelanocytic structures. These spectra were removed from the data set for model creation. When applying the model, orthogonalised spectra with integrated intensity lower than 0.56 were classified as not-melanoma.

#### Outlier detection using PCA

A PCA model was used to identify spectral outliers. The first five principal components, representing 99.9% of the variance in the data set, were used in the model. Outliers were detected by projecting the spectra obtained from lesions on the model. The spectra that could not be explained by the model (i.e. the variance of the residual was larger than 1%) were marked as outliers.

### Histopathological evaluation and exclusion criteria

After Raman measurements, histopathological sections were prepared as part of routine procedure and were evaluated by two expert pathologists dedicated to this study. The final diagnosis, upon agreement, was used as the gold standard for correlation with the Raman measurement, which resulted in annotation of spectra.

All pigmented skin lesions clinically suspicious and excised for diagnostic purpose were eligible for this study. Nonmelanocytic lesions confirmed by histopathology were excluded. Furthermore, the classes of benign melanocytic nevi of which less than five lesions were present in our data set were also excluded.

### Grouping of lesions

**Fig. 2:**
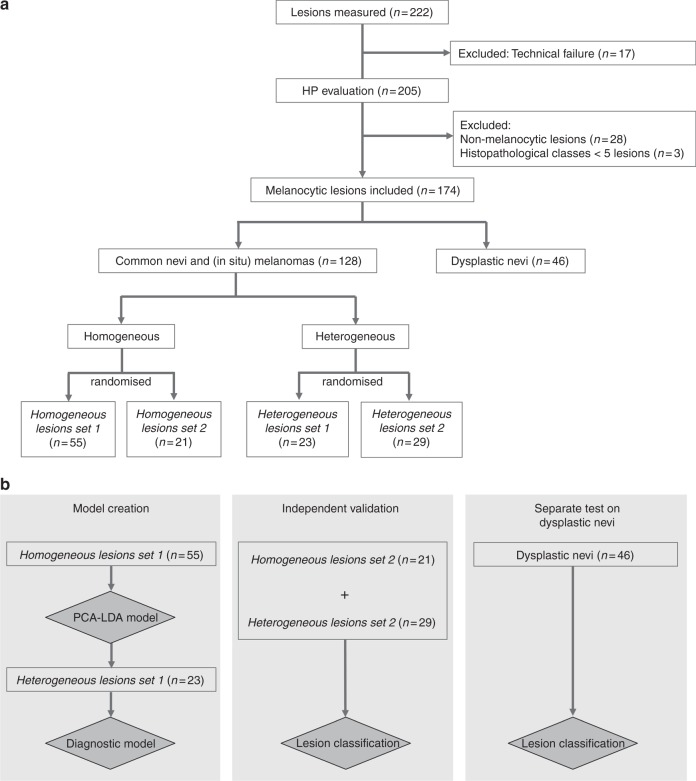
**a** Division of lesions using the histopathological evaluation. The division was used to create the training sets for the diagnostic model (see section “Diagnostic model creation” and **b**). **b** Lesions used for diagnostic model creation (left), lesions used for the independent validation (middle) and dysplastic nevi on which diagnostic model was separately applied (right)

For the purpose of this study, the melanocytic lesions were divided into three groups using histopathological evaluation: (1) homogeneous, (2) heterogeneous, and (3) dysplastic nevi (Fig. 2a). Descriptive details were provided, as the thickness of the lesion, depth of the lesion location in the epidermis/dermis and thickness of stratum corneum. The heterogeneous group comprised: (a) lesions with an uneven distribution of histological components in the most representative H&E slide (e.g. lesion consisted of melanocytic nests surrounded by variable amount of collagen or other nonmelanocytic structures was considered heterogeneous); and (b) lesions whose melanocytic structures were located outside the Raman measurement depth of 300 µm (lesions with a stratum corneum thickness of >300 µm and lesions located at a depth of >300 µm beneath the surface of the skin).

### Diagnostic model creation

#### Creation of training sets for the diagnostic model

From the homogeneous lesions, a subset was randomly selected, referred to as *Homogenous lesions set 1* (Fig. 2a, b). This set was used to create the PCA-LDA model (see below). Also, from the heterogeneous lesions a subset was randomly selected, referred to as *Heterogeneous lesions set 1*. This set was used to define the parameters for the diagnostic model. All histopathologically proven dysplastic nevi were excluded from model creation.

#### PCA-LDA model

A linear discriminant analysis (LDA) model was developed to distinguish melanomas from not-melanoma. The PCA-LDA model was created based on averaged spectra of *Homogeneous lesions set 1*. Principal component analysis (PCA) was performed to reduce the dimensionality of the data prior to LDA modeling. The scores of the spectra on the first three principal components were used as input parameter for the model. The discriminating parameter used as input for the PCA-LDA was a Boolean: “*melanoma*” or “*not-melanoma*”.

#### Parameters for the diagnostic model

The PCA-LDA model was applied to each individual point measurement of *Heterogeneous lesions set 1*. The model yields a probability for each individual point for being melanoma. These probabilities were used to establish the parameters of the diagnostic model. A lesion is classified as melanoma: (1) if two or more individual point measurements within a lesion have a PCA-LDA score higher than 0.35 and/or (2) at least one individual point measurement has a PCA-LDA score greater than 0.8. Otherwise, the lesion is classified as not-melanoma.

### Diagnostic model validation on independent data

The diagnostic model was validated on an independent data set. The independent validation set was comprised of remaining homogeneous and heterogeneous lesions, referred as *Homogeneous lesions set 2* and *Heterogeneous lesions set 2* (Fig. 2a, b). The outcome of the model was a Boolean, “*melanoma*” or “*not-melanoma*”. Specificity was defined as the fraction of correctly predicted negatives (not-melanoma) from the total number of common nevi. Sensitivity was calculated as the fraction of correctly predicted positives (melanoma) from the total number of melanomas.

### Separate test on dysplastic nevi

Because there is no agreement whether dysplastic nevi must be considered benign, they were not included either in the diagnostic model or in the independent validation set. The diagnostic model was separately tested on the dysplastic nevi.

## Results

In total, 222 freshly excised pigmented skin lesions that were clinically suspected of melanoma were measured. From those, a total of 48 were excluded: 17 for technical reasons (spectral artifacts or equipment failure), 28 because these were nonmelanocytic (basal cell carcinoma, seborrheic wart, lichenoid keratosis, dermatofibroma, haemangioma, scar). Table S1 (supplementary material) shows all the lesions included and excluded. In addition, histopathological classes that contained less than five lesions (Spitz nevus, *n* = 2 and combined melanocytic nevus, *n* = 1) were also excluded.

The characteristics of the remaining 174 lesions are summarised in Table 1. Of the 37 melanomas, 22 were in situ (59.4%) and 15 had an average Breslow thickness of 0.89 mm (range 0.2–3.0 mm). Of the total 91 common nevi, 27 were dermal, 43 were compound, 16 were junctional and 5 were blue. The remaining 46 lesions were dysplastic nevi.

**Table 1:** Summary of the lesions included

Histopathological diagnosis	Average age	Sex	Anatomical	Number of lesions	Average Breslow thickness
	(y, range)		Location		(mm, range)
Melanoma					
In situ	58.5 (41–82)	10 female	Head and neck	2	
		12 male	Upper limb	5	
			Trunk	7	
			Lower limb	8	
Invasive	52.9 (29–73)	10 female	Head and neck	2	0.89
		5 male	Upper limb	1	(0.20–3.00)
			Trunk	4	
			Lower limb	8	
Common nevus					
Dermal	43.0 (16–68)	14 female	Head and neck	1	
		13 male	Upper limb	2	
			Trunk	14	
			Lower limb	11	
Compound	46.6 (15−75)	25 female	Head and neck	—	
		18 male	Upper limb	4	
			Trunk	26	
			Lower limb	12	
			Unspecified	1	
Junctional	51.5 (25−82)	10 female	Head and neck	1	
		6 male	Upper limb	2	
			Trunk	11	
			Lower limb	2	
Blue	45.8 (19−87)	3 female	Head and Neck	1	
		2 male	Upper limb	—	
			Trunk	4	
			Lower limb	—	
Dysplastic nevus	47.9 (23−77)	29 female	Head and neck	1	
		17 male	Upper limb	4	
			Trunk	34	
			Lower limb	6	
			Unspecified	1	

Based on the criteria described for lesions classification (see Materials and Methods: Grouping of lesions), 76 lesions were classified as homogeneous, 52 were classified as heterogeneous, and 46 were dysplastic nevi. The two pathologists dedicated to this study had or reached agreement on all diagnosed lesions.

### Diagnostic model creation

A total of 78 lesions were used for model creation (*Homogeneous lesions set 1 and Heterogeneous lesions set 1*). First, the Raman spectra of the *Homogeneous lesions set 1* (55 lesions) were used to create the PCA-LDA model. The Raman spectra of the *Heterogeneous lesions set 1* (23 lesions) were used to define the parameters for the diagnostic model. The histopathological diagnosis of the lesions included in the diagnostic model set are shown in Table 2.

**Table 2:** Histopathological diagnosis of lesions included in the diagnostic model set and in the independent validation set

Histopathological diagnosis		Number of lesions per set		
		Diagnostic model set	Independent validation set	Total
(in situ) Melanoma		20	17	37
Common nevus	Dermal	20	7	27
	Compound	27	16	43
	Junctional	9	7	16
	Blue	2	3	5
Total		78	50	128

Figure S1 (supplementary material) shows the PCA-LDA model discriminant for melanomas vs. common nevi and Figure S2 (supplementary material) shows the loadings of the PCA-LDA model.

### Diagnostic model validation on independent data

*Homogeneous lesions set 2* (21 lesions) *and Heterogeneous lesions set 2* (29 lesions) were used for the independent validation of the diagnostic model. Table 2 shows the histopathological diagnosis of the lesions included in the independent validation set, comprising 17 melanomas (ten in situ and seven melanomas with an average Breslow thickness of 0.42 mm, range 0.2–0.8 mm). Figure S3 (supplementary material) shows the scatter plot of the posterior probability of lesions to belong to melanoma class, using the PCA-LDA model and independent validation. Table 3 shows the contingency table of the diagnostic model validation. A specificity of 43.8 at 100% sensitivity was obtained, specificity defined as the fraction of correctly predicted negatives (not-melanoma) from the total number of common nevi in the independent validation set.

**Table 3:** Contingency table of the diagnostic model validation

		Common nevus	Melanoma	Not predicted (high keratin)
Histopathological diagnosis	Common nevus	14	18	1
	Melanoma	0	16	1

Eighteen common nevi were classified as melanoma, of which seven were compound nevi, five junctional nevi, four dermal nevi and two blue nevi. None of the melanomas was misclassified.

**Fig. 3:**
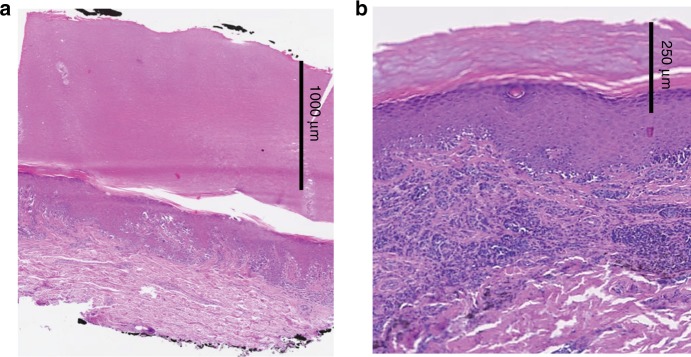
H&E-stained thin tissue sections. **a** Melanoma in situ with a thick stratum corneum (1200 µm); **b** combined melanocytic nevus with a thick stratum corneum (200 µm). N.B.: the Raman spectra of these lesions showed high contribution from keratin and were labeled “Not predicted” by the diagnostic model

Two lesions were identified and labeled “Not predicted” based on the keratin filter. Both lesions were confirmed to have thick stratum corneum by histopathology (one melanoma in situ and one compound nevus, shown in Fig. 3).

### Separate test on dysplastic nevi

The diagnostic model was separately tested on the dysplastic nevi. From the total 46 dysplastic nevi, 73.9% were classified as melanoma (*n* = 34) and 26.1% were classified as benign (*n* = 12).

## Discussion

Melanomas with a Breslow thickness of less than 0.8 mm are curable by surgical excision. In this study, a diagnostic model for melanoma based on Raman spectroscopy was developed and validated on an independent test set. This is the first Raman study addressing thin melanomas, including melanoma in situ. The independent validation set comprised 17 melanomas, of which 10 were in situ. Because detecting melanomas (including the early-stage melanomas and melanomas in situ) is an important clinical goal for disease-free survival, the diagnostic model was optimised for highest possible specificity at a sensitivity of 100%.

To handle the problem of the representative sampling of melanocytes within the heterogeneous lesions, we created a PCA-LDA model using only homogeneous lesions. This ensured that the PCA-LDA model was based on an optimal match between Raman measurements and the reference histopathological diagnosis. The PCA-LDA model discriminant visualises discriminative spectral information between melanomas and common nevi. A higher lipid content in melanoma is the strongest discriminative factor (Figure S1, Supplementary material). The diagnostic model in this study was developed to distinguish melanomas from common nevi suspicious for melanoma.

Dysplastic nevi are melanocytic lesions that present histologically architectural disorder and cytological atypia.^38,39^ Because there is no international consensus about whether dysplastic nevi must be considered benign,^39–45^ dysplastic nevi were not included in the diagnostic model. However, in clinical practice a significant portion of the lesions suspicious for melanoma is dysplastic nevi. Although the dysplastic nevi were not included in the development of the diagnostic model, we have applied the diagnostic model on these lesions as well.

As explained in the Materials and methods section, 9−19 individual point measurements were obtained per lesion. A lesion was classified as melanoma if two or more individual point measurements had a PCA-LDA score higher than 0.35, or if at least one individual point measurement had a PCA-LDA score greater than 0.8. These criteria reflect a melanoma diagnosis based on either a single point measurement with high probability of melanoma or multiple point measurements with moderate probability.

A limitation of this study is the lack of accurate correlation between the individual Raman point measurements and histopathology. We are currently developing a method for reliable and reproducible matching between the origin of individual Raman spectra and histological structures. It is expected that, when applying this method, the accuracy of the diagnostic model that we have developed will be further improved.

In the independent validation, two lesions resulted in the label “Not predicted”. For these lesions, the keratin filter had identified the Raman measurements as nonrepresentative. We consider that labeling nonrepresentative measurements as “Not predicted” is important, as this avoids unnoticed misclassifications.

In this study, the NNT by dermatologists was 6.0 (222 excised lesions suspicious for melanoma, and 37 histopathologically confirmed melanomas). Twenty percent of the excised lesions suspicious for melanoma were dysplastic nevi. To calculate the NNT based on Raman diagnosis, 13 randomly selected dysplastic nevi were added to the validation set, so that this set also comprised 20% of dysplastic nevi. If the Raman instrument were used as an add-on to diagnose the dermatologist-selected lesions, the estimated NNT would be 2.7 (43 lesions tested positive by Raman spectroscopy and a total 16 histopathologically confirmed melanoma). This would represent detecting all thin melanomas and reducing the number of unnecessary excisions by more than twofold, when comparing with the current clinical practice. There are indications that dysplastic nevi are associated with an increased risk of developing melanoma,^40,46–48^ which is suggestively supported by our results shown in the subsection “Separate test on dysplastic nevi”.

The study was conducted on a tertiary referral center by highly specialised dermatologists. The potential reduction of NNT to 2.7 can be even more significant when considering the NNT reported for nonspecialists (NNT = 20–30).^5−13^ To optimise the diagnostic model for different settings the current model needs to be expanded to include more types of suspicious skin lesions, considering the multitude of skin lesions that dermatologists and general practitioners have suspicions on in a more generalised clinical practice.

We have demonstrated that an accurate diagnosis of thin melanoma, including melanoma in situ, can be made based on Raman spectroscopy. This signifies an important step towards accurate and objective clinical diagnosis of melanoma.

## Electronic supplementary material

Figure S1

Figure S2

Figure S3

Table S1

## **Availability of data and material:**

The data generated during this study are available from the corresponding author on reasonable request.
