# Supplementary figures and images for: Improving clinical diagnosis of early-stage cutaneous melanoma based on Raman spectroscopy
Source: Br J Cancer. 2018 Nov 9;119(11):1339–46. doi: 10.1038/s41416-018-0257-9 (PMC6265324; doi:10.1038/s41416-018-0257-9)

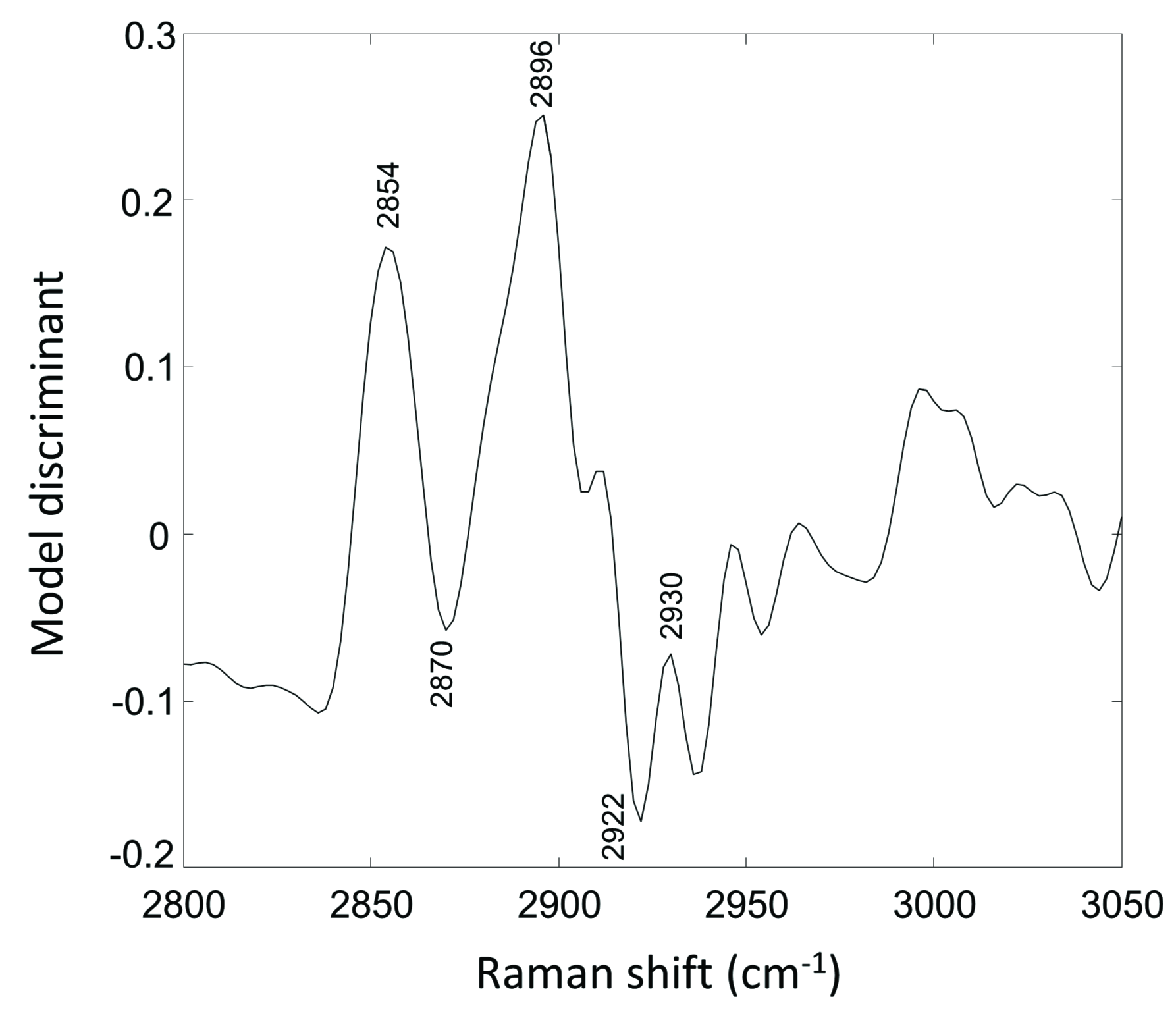

Supplement: Supplementary file 1 — Figure S1 [file 41416_2018_257_MOESM1_ESM.tif]
